# Brain-derived neurotrophic factor serum levels following ketamine and esketamine intervention for treatment-resistant depression: secondary analysis from a randomized trial

**DOI:** 10.47626/2237-6089-2021-0298

**Published:** 2023-02-02

**Authors:** Ana Teresa Caliman-Fontes, Gustavo C. Leal, Fernanda S. Correia-Melo, Camilla S. Paixao, Michelle S. Carvalho, Ana Paula Jesus-Nunes, Flavia Vieira, Guilherme Magnavita, Igor D. Bandeira, Rodrigo P. Mello, Graziele Beanes, Samantha S. Silva, Mariana Echegaray, Lucas P. Carvalho, Paulo Machado, Aline S. Sampaio, Taiane de A. Cardoso, Flávio Kapczinski, Acioly L. T. Lacerda, Lucas C. Quarantini

**Affiliations:** 1 Faculdade de Medicina da Bahia UFBA Salvador BA Brazil Faculdade de Medicina da Bahia, Universidade Federal da Bahia (UFBA), Salvador, BA, Brazil.; 2 Departamento de Neurociências e Saúde Mental Faculdade de Medicina da Bahia UFBA Salvador BA Brazil Laboratório de Neuropsicofarmacologia, Departamento de Neurociências e Saúde Mental, Faculdade de Medicina da Bahia, UFBA, Salvador, BA, Brazil.; 3 Serviço de Psiquiatria Hospital Universitário Professor Edgard Santos UFBA Salvador BA Brazil Serviço de Psiquiatria, Hospital Universitário Professor Edgard Santos, UFBA, Salvador, BA, Brazil.; 4 Programa de Pós-Graduação em Medicina e Saúde Faculdade de Medicina da Bahia UFBA Salvador BA Brazil Programa de Pós-Graduação em Medicina e Saúde, Faculdade de Medicina da Bahia, UFBA, Salvador, BA, Brazil.; 5 Laboratório de Pesquisas Clínicas Instituto Gonçalo Moniz Fiocruz Salvador BA Brazil Laboratório de Pesquisas Clínicas, Instituto Gonçalo Moniz, Fiocruz, Salvador, BA, Brazil.; 6 Serviço de Imunologia UFBA Salvador BA Brazil Serviço de Imunologia, UFBA, Salvador, BA, Brazil.; 7 Laboratório Interdisciplinar de Neurociências Clínicas Universidade Federal de São Paulo São Paulo Brazil Laboratório Interdisciplinar de Neurociências Clínicas, Universidade Federal de São Paulo, São Paulo, Brazil.; 8 Department of Psychiatry and Behavioural Neurosciences McMaster University Hamilton ON Canada Department of Psychiatry and Behavioural Neurosciences, McMaster University, Hamilton, ON, Canada.; 9 Mood Disorders Program Department of Psychiatry and Behavioural Neurosciences McMaster University Hamilton ON Canada Mood Disorders Program, Department of Psychiatry and Behavioural Neurosciences, McMaster University, Hamilton, ON, Canada.

**Keywords:** BDNF, biochemical markers, major depressive disorder, NMDA antagonists, neurotrophins

## Abstract

**Objectives:**

Evidence suggests that ketamine’s influence on brain-derived neurotrophic factor (BDNF) might be involved in its mechanism of rapid antidepressant action. We aimed to evaluate the differential impact of ketamine and esketamine on serum BDNF levels and its association with response patterns in treatment-resistant depression (TRD).

**Methods:**

Participants (n = 53) are from a randomized, double-blind clinical trial comparing the efficacy of single-dose ketamine (0.5mg/kg, n = 27) and esketamine (0.25mg/kg, n = 26) in TRD. Depression severity was assessed before and 24 hours, 72 hours, and 7 days after the intervention, using the Montgomery-Åsberg Depression Rating Scale (MADRS). Blood samples were collected before infusion, 24 hours, and 7 days afterwards.

**Results:**

There were no significant changes in BDNF levels at post-infusion evaluation points, and no difference in BDNF levels comparing ketamine and esketamine. Both drugs exhibited similar therapeutic effect. There was no association between BDNF levels and response to treatment or severity of depressive symptoms.

**Conclusion:**

There was no significant treatment impact on BDNF serum levels – neither with ketamine nor esketamine – despite therapeutic response. These results suggest that ketamine or esketamine intervention for TRD has no impact on BDNF levels measured at 24 hours and 7 days after the infusion. This clinical trial is registered on the Japan Primary Registries Network: UMIN000032355.

## Introduction

Major depressive disorder (MDD) is a major social and economic burden, being the leading cause of incapacity worldwide.^1^ Traditional antidepressants require several weeks to exert therapeutic effect, and only one third of patients with MDD achieve remission after multiple treatments.^2^ The N-methyl-D-aspartate (NMDA) receptor antagonist, ketamine, has emerged as an alternative with rapid antidepressant action for patients who did not respond to monoaminergic antidepressants.^3 - 5^ Ketamine is a racemic mixture of two enantiomers: S(+)-ketamine (or esketamine) and R(-)-ketamine (or arketamine), with the S(+) stereoisomer presenting a greater affinity for the NMDA receptor than R(-)-ketamine.^6^ Intranasal esketamine was the first FDA-approved rapid antidepressant for treatment-resistant depression (TRD),^7^ and intravenous racemic ketamine was considered to have Level 1 evidence for efficacy in the latest update of the Canadian Network for Mood and Anxiety Treatment (CANMAT) guidelines for MDD.^8^

Evidence links the antidepressant action of ketamine to increases in brain-derived neurotrophic factor (BDNF) levels.^9 - 11^ BDNF is a growth factor from the family of neurotrophins, which plays a key role in development, differentiation, and survival of neurons, as well as in neuroplasticity.^12 , 13^ There is mounting evidence indicating involvement of BDNF in stress response and in the pathophysiology of mood disorders.^14 , 15^ In depressive patients, BDNF levels are reduced in some brain regions and in peripheral blood^16 - 19^ and seem to correlate with improvement of depression.^20^ Animal studies even suggest that BDNF is necessary for the efficacy of antidepressants, including ketamine.^21^

Having a valid biomarker to guide treatment of mood disorders has always been longed-for in Psychiatry, with BDNF as one of the most promising candidates. Assessing peripheral BDNF in the context of treatment with different forms of ketamine can not only contribute to the elucidation of its role as a biomarker, but also to clarification of the mechanism of action of ketamine, since the racemate and esketamine may have different pharmacological effects over BDNF. The main objective of the present study was to explore the differential impact of a single dose of ketamine or esketamine on BDNF serum levels in TRD subjects. We also aimed to evaluate the association of BDNF levels with response patterns in TRD, including the utility of baseline BDNF for predicting response with ketamine and esketamine treatment.

## Methods

### Study design

This study was conducted as a secondary analysis of data from a randomized, double-blind clinical trial designed to compare the efficacy of ketamine and esketamine for TRD, registered on the Japan Primary Registries Network: UMIN000032355. The study was conducted at the University Hospitals of both the Federal University of Bahia (UFBA) and Federal University of Sao Paulo (UNIFESP), in Brazil. Details on sample calculation, other methodological information and primary outcome results can be found in the study protocol published in 2018 and the efficacy trial published in 2019.^22 , 23^

### Participants

Participants were aged 18 years or older, with a diagnosis of MDD by DSM-IV criteria and failure to respond to at least one adequate antidepressant treatment. Major exclusion criteria included: current treatment with electroconvulsive therapy; dementia, intellectual disability, or psychosis; current use of illicit drugs; and cardiovascular decompensation. Subjects were on oral antidepressant treatment and did not have to discontinue current medications, which should remain unchanged from 15 days before intervention until the end of the follow-up period.

### Intervention

Intervention consisted of a single intravenous infusion of either 0.5mg/kg of ketamine racemate (ketamine hydrochloride), or 0.25mg/kg of esketamine (S(+)-ketamine or dextroketamine hydrochloride). Both drugs were diluted in saline and administered intravenously for 40 minutes. Participants were randomized into esketamine and ketamine groups at a 1:1 ratio, using electronic randomization software.

### Outcomes

Depression severity was assessed with the Montgomery-Åsberg Depression Rating Scale (MADRS) before the intervention and then 24 hours, 72 hours, and 7 days after. Therapeutic response was defined as a 50% or greater reduction in MADRS score compared to baseline. For assessment of BDNF levels, blood samples were collected right before the infusion, then 24 hours and 7 days after. All blood collections occurred in the afternoon. At baseline assessment, participants fasted for at least 4 hours before blood collection (required for safe administration of the study drug), but a fasting period was not standardized for the subsequent time points.

Blood samples were collected in separation gel tubes, centrifuged to obtain serum, and then aliquoted and stored at -70°C. The test was performed in the Immunology Laboratory at the University Hospital of the Federal University of Bahia. BDNF concentrations were measured by enzyme-linked immunosorbent assay (ELISA by DuoSet; R&D Systems) following the manufacturer’s instructions. Absorbance was read with a Multiskan FC plate reader (Thermo Fischer Scientific Inc., USA) at 450 nm. Results are expressed in pg/mL.

### Statistical analysis

A general linear model (GLM) of repeated measures was used to assess changes in MADRS scores and BDNF levels over time and to evaluate possible differences between the two intervention groups. Differences in BDNF levels over time between responders and non-responders were analyzed with the same model. We used Spearman’s rank correlation coefficients to evaluate the relationship between serum BDNF levels and MADRS scores at each time point, and between changes in BDNF and MADRS from baseline to 24 hours. Finally, we used a multiple linear regression model to assess the ability of BDNF levels to predict changes in MADRS scores. We adopted the value of *α* = 0.05 as the threshold for statistical significance. Statistical analysis was conducted using IBM SPSS Statistic Base software (version 21.0).

### Ethical considerations

Independent Institutional Review Boards approved the study protocol and its amendments (Professor Edgard Santos University Hospital, UFBA, Number: 46657415.0.0000.0049 and São Paulo Hospital, UNIFESP, Number: 46657415.0.3001.5505). The study was conducted in accordance with the Declaration of Helsinki, consistent with Good Clinical Practice guidelines and applicable regulatory requirements. All participants signed written informed consent before any study procedures were initiated.

### Data accessibility statement

The data that support the findings of this study are available for public access at http://dx.doi.org/10.17632/3rbpmwxrm5.1.^24^

## Results

### Participant flow

**Figure 1:**
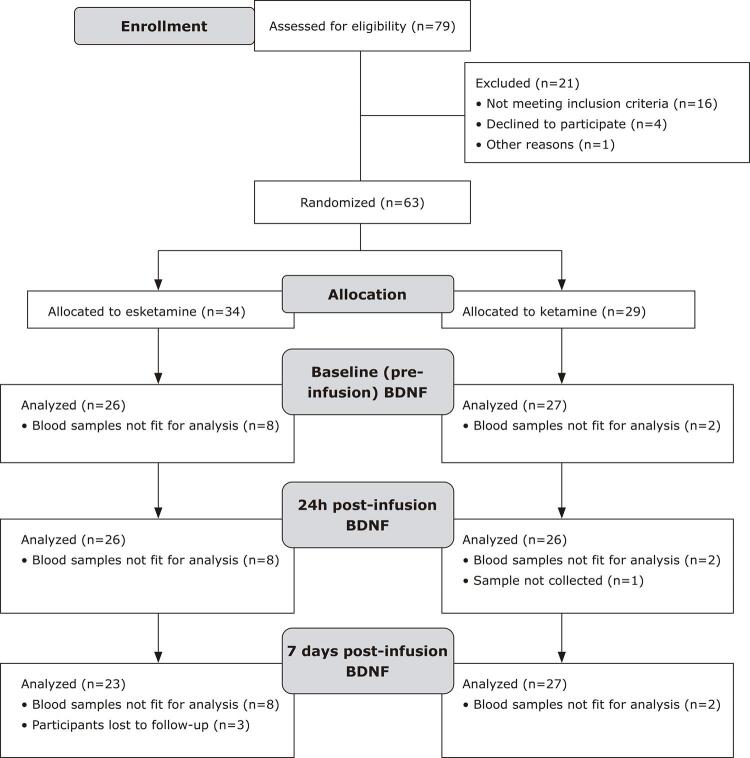
Flowchart illustrating study participation. BDNF = brain-derived neurotrophic factor.

Participants were recruited between March 2017 and June 2018. A total of 63 patients were enrolled on the present trial; a participants’ flowchart can be seen in Figure 1 . Baseline characteristics were well balanced between groups and can be consulted in Table 1 .

**Table 1:** Demographic and clinical characteristics of participants divided by intervention groups

Characteristic	Esketamine (n = 26)		Ketamine (n = 27)	

	Mean	SD	Mean	SD
Age (years)	47.9	14.9	49.2	14.8
Duration of current depressive episode (months)	31	71.7	22	40.9
Age at first depressive episode (years)	34.6	16.9	34.8	18.4
Number of depressive episodes	7.5	6.5	5.5	5.2
Number of therapeutic failures	3.1	1.3	4.5	2.1
Baseline MADRS score	32	9.52	32.93	5.43
	
	**n**	**%**	**n**	**%**
	
Female gender	15	57.7	17	68
Personal history of suicide attempt(s)	12	46.2	9	33.3
Current generalized anxiety disorder	5	19.2	7	26.9
Current or previous panic disorder	8	30.8	7	26.9
Current post-traumatic stress disorder	1	3.8	2	7.7
Current antidepressant treatment*				
Monotherapy with SSRIs	4	15.4	3	11.1
Monotherapy with SNRIs	2	7.7	1	3.7
Monotherapy with other AD^†^	0	0	1	3.7
AD combination	2	7.7	4	14.8
AD augmentation	18	69.2	15	55.6
Other treatment^‡^	0	0	2	7.4

AD = antidepressant; MADRS = Montgomery-Åsberg Depression Rating Scale; SD = standard deviation; SNRIs = Serotonin-norepinephrine reuptake inhibitors; SSRIs = Selective serotonin reuptake inhibitors.

* Information on the current treatment regimen was missing for one patient.

^†^ The single participant in this category was on monotherapy with trazodone.

^‡^ Both participants were on monotherapy with quetiapine.

### Therapeutic response

**Figure 2:**
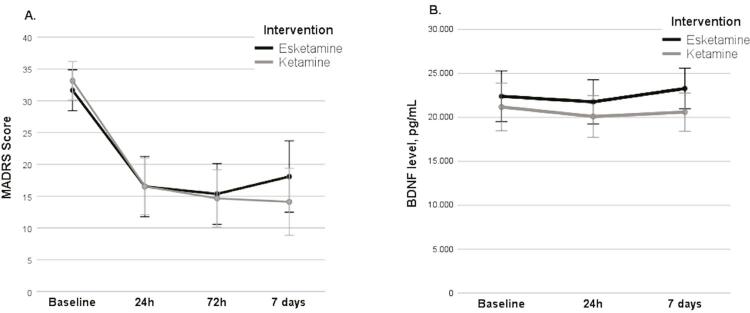
Differences between intervention groups (Esketamine, n = 26, and Ketamine, n = 27) over time. A) MADRS mean scores at the different time points, B) BDNF serum levels at the different time points. Error bars represent 95% confidence intervals of means. BDNF = brain-derived neurotrophic factor; MADRS = Montgomery-Åsberg Depression Rating Scale.

There was a significant change in MADRS scores across the evaluation points (F_3, 141_ = 102.272, p < 0.001, partial η^2^ = 0.685), and no significant difference between intervention groups (F_1, 47_ = 0.076, p = 0.784, partial η^2^ = 0.002). There was also no significant interaction between MADRS scores and intervention group (F_3, 141_ = 1.989, p = 0.142, partial η^2^ = 0.041) ( Figure 2A ). Thus, the therapeutic effect on depressive symptoms was similar for esketamine and ketamine in our sample.

Response ratios were similar for both drugs at all time points. At 24 hours after treatment, 53.8% of the group receiving esketamine and 51.9% of the ketamine group were responders ( *d* = 1.9% [95%CI -0.23 to 0.27]). At 72 hours, the proportions of responders were 50% and 57.7% for esketamine and ketamine ( *d* = -7.7% [95%CI -0.33 to 0.19]), respectively, and at 7 days they were 50% and 63% ( *d* = -13% [95%CI -0.37 to 0.13]).

### Effect of treatment on BDNF levels

Mean values of BDNF for the intervention groups at each timepoint can be consulted in Table 2 . We found there was no significant change in BDNF across the three measurement points (F_2, 94_ = 0.536, p = 0.587, partial η2 = 0.011). There was also no significant difference in BDNF levels between intervention groups (F_1, 47_ = 2.118, p = 0.152, partial η2 = 0.043), and no significant interaction between time and intervention group (F_2, 94_ = 0.260, p = 0.771, partial η2 = 0.006) ( Figure 2B ).

**Table 2:** Serum BDNF levels (pg/mL) measured at different time-points by intervention groups

Time-point	Esketamine (n = 26)	Ketamine (n = 27)	Mean difference (95%CI)	p-value
Baseline*	22,092 (5,880)	21,180 (7,354)	912 (-2,768; 4,593)	0.621
24 hours*	21,871 (6,042)	20,103 (5,685)	1,769 (-1,499; 5,036)	0.282
7 days*	23,292 (4,431)	20,600 (6,157)	2,691 (-409; 5,792)	0.087

BDNF = brain-derived neurotrophic factor; 95%CI = 95% confidence interval.

* Data are expressed as mean (standard deviation).

### Treatment response and BDNF levels

With the GLM repeated measures of BDNF over time, but with both intervention group and 24h response status as between-subjects factors, we did not find a significant interaction (F_1,45_ = 0.27, p = 0.869, partial η^2^ = 0.001), demonstrating that being a responder or not did not impact BDNF levels changes over time in either treatment group.

As for the predictive model of depressive symptoms improvement 24 hours post-infusion, a multiple linear regression demonstrated that BDNF levels at baseline ( *t* = 0.475, p = 0.637), 24 hours ( *t* = -0.607, p = 0.547), and 7 days ( *t* = 0.293, p = 0.770) were not associated with changes in MADRS scores, and the model was not significant ( *R* ^2^ = 0.013). The same was observed at 7 days post-infusion ( *R* ^2^ = 0.021), in which BDNF levels at baseline ( *t* = 0.750, p = 0.457), 24 hours ( *t* = -0.227, p = 0.821), or 7 days ( *t* = -0.790, p = 0.433) were not predictors of MADRS decrease.

### BDNF levels and severity of depression

Correlation between baseline MADRS scores and serum BDNF levels was not significant ( *ρ* = 0.055, p = 0.694), or at any of the other time points. Also, no correlation was found between absolute MADRS and BDNF variation from baseline to 24 hours (ρ = 0.079, p = 0.576).

## Discussion

We found no significant alteration in BDNF levels 24 hours and 7 days after the infusion of ketamine or esketamine, compared to baseline. We also did not find any association between BDNF levels and response to treatment. As far as we know, this is the first study directly comparing the effects of ketamine and esketamine on BDNF levels in TRD patients. Liu et al.^25^ measured BDNF levels after low-dose ketamine and esketamine, but the drugs were administered right before surgery for breast cancer, and patients had only mild to moderate depression, not necessarily treatment-resistant depression.

The present study design cannot rule out early and transient changes in peripheral BDNF levels after ketamine and esketamine administration. In animals, one study suggested ketamine raises levels of BDNF mRNA in rat hippocampal neurons within 30 minutes, sustained for at least 24 hours,^26^ but another demonstrated an increase in BDNF at 30 minutes, but not at 24 hours post-infusion.^27^ In humans, data regarding speed and duration of BDNF changes vary greatly. Previous studies demonstrating changes in BDNF levels after ketamine administration analyzed blood samples collected within 230 or 240 minutes post-infusion.^10 , 28^ However, other studies did not find early (230 minutes) changes in BDNF levels after treatment with ketamine.^29 , 30^ Moreover, this last cited recent study, conducted by Medeiros et al., also did not detect changes in BDNF levels at 24 or 72 hours post-infusion, which is accordance with our results. On the other hand, a study comparing ketamine and electroconvulsive therapy showed increased levels of BDNF starting only one week after the first infusion of ketamine.^31^ Liu et al.^25^ found elevated BDNF levels from three days to one month after ketamine and esketamine administration in depressed subjects, but, as stated before, the infusions occurred before surgery, and the effects of the procedure cannot be discarded. Therefore, the optimal period for assessment of peripheral BDNF is still unclear.

In our sample, BDNF levels did not correlate with depression severity. Similar findings were obtained in a study with 962 depressed patients,^19^ but they contrasted with other studies that found a negative correlation.^10 , 18 , 32^ In our study, lower levels of BDNF at baseline were not associated with a lack of therapeutic response, a finding that is in line with one previous study.^33^

We chose to measure serum BDNF instead of plasma levels because it is more stable, reproductible,^34^ and considered more reliable.^35^ The BDNF concentration in serum is about 100 times higher than in plasma, which is probably due to release of BDNF by platelets during the coagulation process.^36 , 37^ Therefore, measuring peripheral BDNF in serum, compared to plasma, might influence the assessment sensibility, since the significant platelet pool of BDNF – released after platelet activation – could mask possible discrete acute variations in this neurotrophin.

There is evidence that alterations in BDNF serum levels in humans and rats primarily reflect processes occurring in megakaryocytes and platelets, not necessarily reflecting neuronal levels.^38^ Furthermore, a recent study in animal models has called into question the correlation between central and peripheral BDNF levels and their use as a biomarker for ketamine’s effects.^39^

Regarding a possible differential effect of the enantiomers of ketamine, previous studies in murine models highlighted R(-)-ketamine as having a longer-lasting antidepressant action when compared to S(+)-ketamine,^40^ suggesting an impact on BDNF levels and BDNF-TrkB signaling as a mediator of its longer-lasting effect.^41^ In the present study, however, there was no differential impact of racemic ketamine or esketamine on BDNF levels at the timepoints evaluated.

Our study has some limitations: not being specifically designed to evaluate BDNF levels; the absence of a control group receiving placebo; and non-standardized concomitant medication use, which, on the other hand, may better reflect the naturalistic scenario of clinical practice.

## Conclusion

In conclusion, serum measurements of BDNF levels 24 hours and 7 days after treatment were not different between ketamine and esketamine groups, and BDNF levels were not associated with treatment response to either drug. Possibly, this negative result, rather than discrediting the role of BDNF in the antidepressant mechanism of action of ketamine, suggests that these post-infusion times may not be the most appropriate for peripheral BDNF measurement. Further studies with larger samples will be needed to clarify this possible differential effect of ketamine forms. Additionally, we suggest measuring BDNF within a shorter period after ketamine infusion since it might be a more suitable window for detection of variations in this neurotrophin.
